# Intraoperative and Short‐Term Postoperative Outcomes Associated With Transoral Robotic Surgery: A Protocol for a Scoping Review

**DOI:** 10.1111/aas.70052

**Published:** 2025-04-28

**Authors:** Amanda Albæk Larsen, Magnus Fuhr Hovind, Emma Atsuko Tsuchiya, Anders Christensen, Johan Heiberg, Mette Krag, Camilla Strøm

**Affiliations:** ^1^ Department of Anaesthesia Centre of Head and Orthopaedics, Rigshospitalet Copenhagen Denmark; ^2^ Department of Otorhinolaryngology, Head and Neck Surgery and Audiology Rigshospitalet Copenhagen Denmark; ^3^ Department of Clinical Medicine University of Copenhagen Copenhagen Denmark

**Keywords:** anesthesia, robotic surgical procedures, scoping review, treatment outcome

## Abstract

**Background:**

Transoral robotic surgery has been introduced into clinical practice as a minimally invasive procedure for the treatment of both malignant and benign conditions within the field of otorhinolaryngology. While the long‐term outcomes of this innovative surgical technique have been extensively studied, evidence regarding intraoperative and short‐term postoperative outcomes remains limited. This scoping review aims to systematically outline and evaluate the current evidence on perioperative outcomes.

**Methods:**

This protocol has been developed in accordance with the Preferred Reporting Items for Systematic Review and Meta‐Analysis Protocol and will adhere to the PRISMA Extension for Scoping Reviews guidelines for reporting. A structured “Population, Intervention, Comparator, and Outcome” framework will guide the definition of search criteria. A comprehensive search will be conducted in The Cochrane Library, MEDLINE, and Embase databases. The study selection and screening process will be performed independently by two reviewers, with consensus reached in cases of disagreement. All identified intraoperative and short‐term postoperative outcomes of transoral robotic surgery will be analyzed and described.

**Discussion:**

The aim of this scoping review is to provide a comprehensive overview of the existing evidence on intraoperative and short‐term postoperative outcomes associated with transoral robotic surgery. This will help identify gaps in the literature and inform future clinical practice and research.

## 1 Introduction

Transoral robotic surgery (TORS) has emerged as a minimally invasive surgical technique that offers improved access to the pharynx compared to conventional surgical approaches. This innovative method enables precise resection of lesions in the pharynx without the need for more invasive procedures such as mandibulotomy or pharyngotomy, which are associated with substantial surgical morbidity [1].

Despite the growing adoption of TORS in clinical practice, there is a lack of comprehensive understanding regarding intraoperative management and short‐term outcomes, including anesthesia‐related considerations and postoperative recovery. The absence of a clear evidence‐based overview highlights the need for this scoping review to systematically map the current knowledge and identify gaps in the literature to guide future research and clinical practice [2].

## 2 Objectives

The aim of this scoping review is to provide a comprehensive overview of the existing evidence on intraoperative and short‐term postoperative outcomes associated with TORS.

## 3 Review Questions

The foundation of this investigation builds on the following review questions:

- What has been the primary anesthetic approach to patients undergoing TORS?
- Which perioperative complications are the most dominant?
- Which short‐term outcomes are typically seen after TORS?
- Can any general recommendations for a safe anesthesiologic approach for TORS procedures be extracted from current literature?

All the above‐mentioned questions should be in comparison to other medical techniques apart from TORS.

## 4 Methods

This protocol has been designed according to the Preferred Reporting Items for Systematic reviews and Meta‐Analyses (PRISMA) [3]. The scoping review will be reported according to the PRISMA extension for scoping reviews (PRISMA‐ScR) guideline [4].

### 4.1 Study Design

A population, intervention, comparator, and outcome‐based (PICO) approach will define our eligibility criteria [5].

*Population*: We will include adult patients undergoing TORS for any reason. Exclusion criteria: non‐robotic transoral surgery, pediatric patients, studies focused solely on long‐term outcomes (defined by outcomes measured > 30 days from day of surgery), cadaver studies.

*Intervention*: TORS as a minimally invasive surgical approach.

*Comparator*: Non‐robotic surgery, radiation therapy, or no comparison group.

*Outcomes*: All reported intraoperative and short‐term postoperative outcomes will be described. We will focus on outcomes within the first 30 days.

Studies of cadavers, animals, and children, and those that focus solely on long‐term outcomes will be excluded.

Two review authors will independently assess all titles and abstracts for eligibility. Trials that obviously do not match inclusion criteria will be excluded. In case of disagreements, a third review author will be involved to arbitrate. Trials that initially were deemed to be relevant, but subsequently are excluded, will be listed with the main reason for their exclusion. The reference lists of included papers will be screened manually. Finally, the search strategy will be added in an appendix of the scoping review to clarify the scientific method used. The selection process will be reported in a PRISMA flowchart and added in the appendix.

### 4.2 Data Extractions and Management

Two review authors will extract information from the included studies using a predesigned data extraction form. The information will include trial characteristics, participant characteristics, indication for TORS, type of comparator (for comparative studies), anesthetic management, and outcomes.

### 4.3 Strategy of Data Synthesis

We will present our data descriptively with the intention of summarizing outcomes systematically. We aim to thematize outcomes in groups and present these. No meta‐analysis will be conducted.

## 5 Discussion

Conducting a thorough investigation of the existing literature will help to synthesize the current evidence and identify future research questions. This scoping review is strengthened by its foundation in a systematic search strategy and its adherence to established methodologies, including a PICO‐based selection of studies and compliance with PRISMA. However, potential limitations of the review include inherent biases in the framing of the research questions, the possibility of insufficient existing literature to comprehensively address the topic, and the reliance on a descriptive approach to categorize outcomes, which may limit the depth of the analysis.

## 6 Conclusion

The scoping review will summarize the current literature on the intraoperative and short‐term postoperative outcomes in relation to TORS.

## Author Contributions

Amanda Albæk Larsen conceptualized the study, developed the protocol, conducted the literature search in collaboration with Emma Atsuko Tsuchiya, and performed the study selection and screening together with Magnus Fuhr Hovind. Amanda drafted the manuscript, which was critically revised and refined in collaboration with all co‐authors. All authors contributed to the final version of the manuscript and approved it for submission.

## Conflicts of Interest

The authors declare no conflicts of interest.

## Data Availability

Research data are not shared.
